# Impact of human papillomavirus infection on semen parameters and reproductive outcomes

**DOI:** 10.1186/s12958-021-00840-y

**Published:** 2021-10-09

**Authors:** Hana Jaworek, Vladimira Koudelakova, Ivana Oborna, Blazena Zborilova, Jana Brezinova, Dagmar Ruzickova, Jana Vrbkova, Pavla Kourilova, Marian Hajduch

**Affiliations:** 1 Institute of Molecular and Translational Medicine, Faculty of Medicine and Dentistry, Palacky University Olomouc, Hnevotinska 1333/5, 779 00 Olomouc, Czech Republic; 2 Fertimed Ltd., Boleslavova 2, 776 00 Olomouc, Czech Republic; 3 SpermBank International, Katerinska 13, 779 00 Olomouc, Czech Republic; 4 Arleta IVF Ltd., Komenskeho 702, 517 41 Kostelec nad Orlici, Czech Republic

**Keywords:** Human papillomavirus, Semen, Penile swab, Infertility, Sperm donor

## Abstract

**Background:**

Human papillomavirus (HPV) has been shown to adversely affect human reproduction. We aimed to evaluate the prevalence of human papillomavirus (HPV) infection in men and its correlation with semen parameters and reproductive outcomes.

**Methods:**

Semen samples and penile swabs were collected from potential sperm donors (SD, *n* = 97) and male partners of infertile couples (IM, *n* = 328). The presence of HPV DNA in semen samples and penile swabs was analyzed. Associations between hrHPV positive status and fertility outcomes as well as socio-behavioral and health characteristics were evaluated using the R software package.

**Results:**

High-risk HPV (hrHPV) genotypes were detected in 28.9% of SD and 35.1% of IM (*P* = 0.312). Penile swabs were more frequently positive for hrHPV genotypes than semen samples in both IM (32.3% vs. 11.9%, *P* < 0.001) and SD (26.8% vs. 6.2%, *P* = 0.006).

Men with hrHPV positive semen samples had lower semen volume (median volume 2.5 ml vs. 3 ml, *P* = 0.009), sperm concentration (median concentration 16 × 10^6^/ml vs. 31 × 10^6^/ml, *P* = 0.009) and total sperm count (median count 46 × 10^6^ vs. 82 × 10^6^, *P* = 0.009) than men with hrHPV negative samples. No association was identified between penile hrHPV status and semen parameters.

**Conclusions:**

Our findings indicate that penile HPV infection is common in both potential sperm donors and men from infertile couples. Although HPV positivity is higher in penile swabs, only HPV infection in semen samples affects sperm parameters. However, there was no association between hrHPV positivity in semen and fertility outcomes including abortion rate.

**Supplementary Information:**

The online version contains supplementary material available at 10.1186/s12958-021-00840-y.

## Background

Human papillomaviruses (HPVs) are the causative agents of a common sexually transmitted disease that preferentially infects squamous epithelial cells [1] and are also an important factor in carcinogenesis in both men and women [2, 3]. Many studies have focused on HPV-associated diseases in women, but there is comparatively little data on male infection. The estimated prevalence of HPV infection in males ranges from 1.3 to 72.9% depending on the anatomic site of specimen collection and testing method [4, 5]. While male HPV infections may be associated with lower mortality and morbidity than HPV infections in women, they still warrant investigation due to their association with genital warts, penile, anal, and oropharyngeal cancer, and the high risk of transmission to sexual partners [6, 7].

HPV can be found along the entire male and female genital tract as well as in semen, where it binds to the sperm head and significantly reduces sperm motility [5, 8]. Moreover, *Foresta* et al. showed that HPV infected spermatozoa can penetrate the oocyte [9], and multiple in vitro studies have indicated that HPV can adversely affect early embryonic development [10–12]. Consequently, there is growing interest in the impact of HPV on male fertility. Several studies have shown that HPV infection can negatively influence pregnancy rates during assisted reproduction treatment (ART) and increase abortion rates in both spontaneous pregnancies and pregnancies after ART [13–15].

In this study we investigated the prevalence of HPV in semen samples and penile swabs from potential sperm donors (SD) and male partners from couples treated for infertility (IM), and its impact on semen parameters and fertility outcomes.

## Methods

### Study design and inclusion criteria

**Fig. 1:**
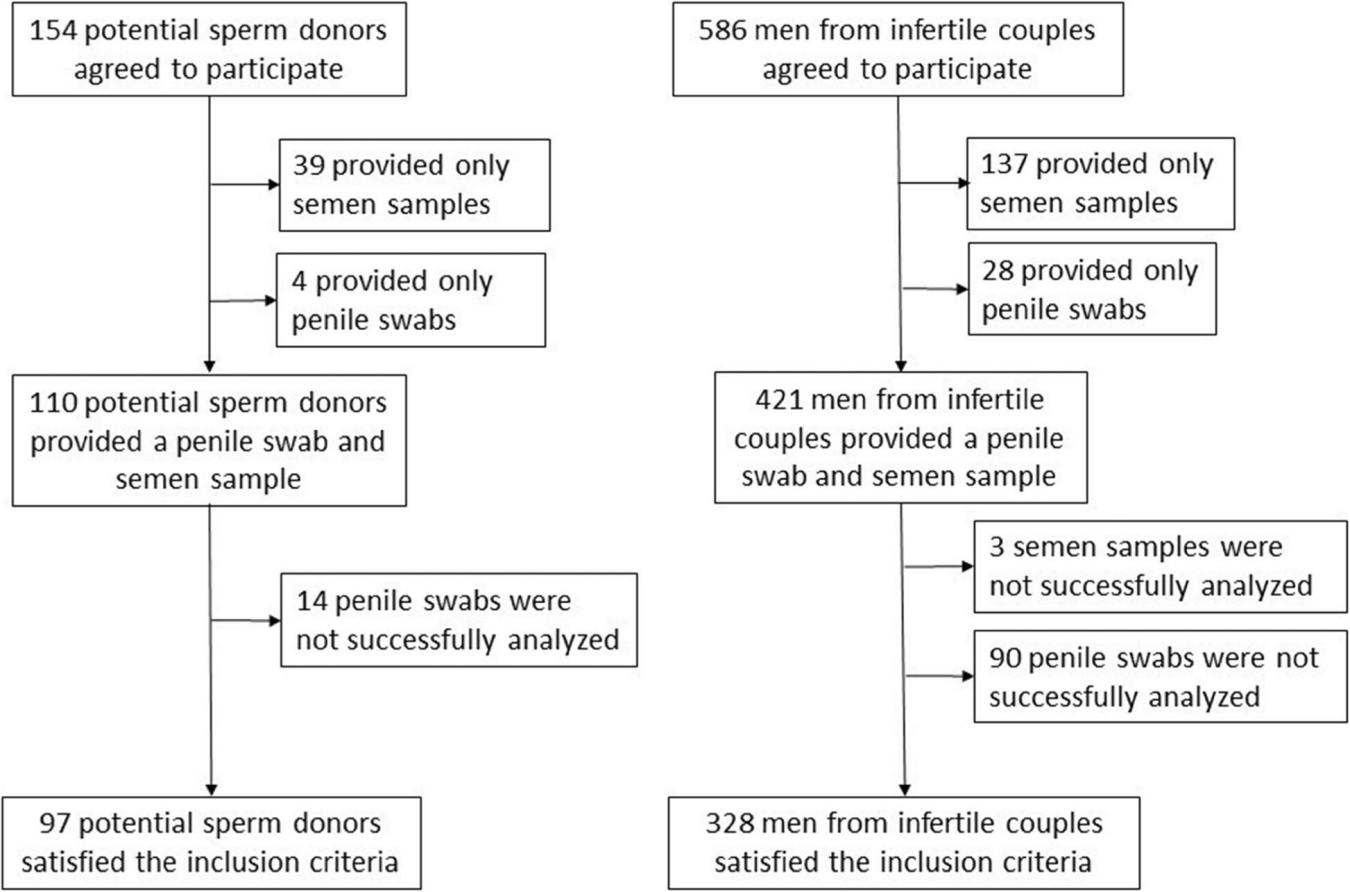
Study flowchart - male partners from couples treated for infertility and potential sperm donors

**Fig. 2:**
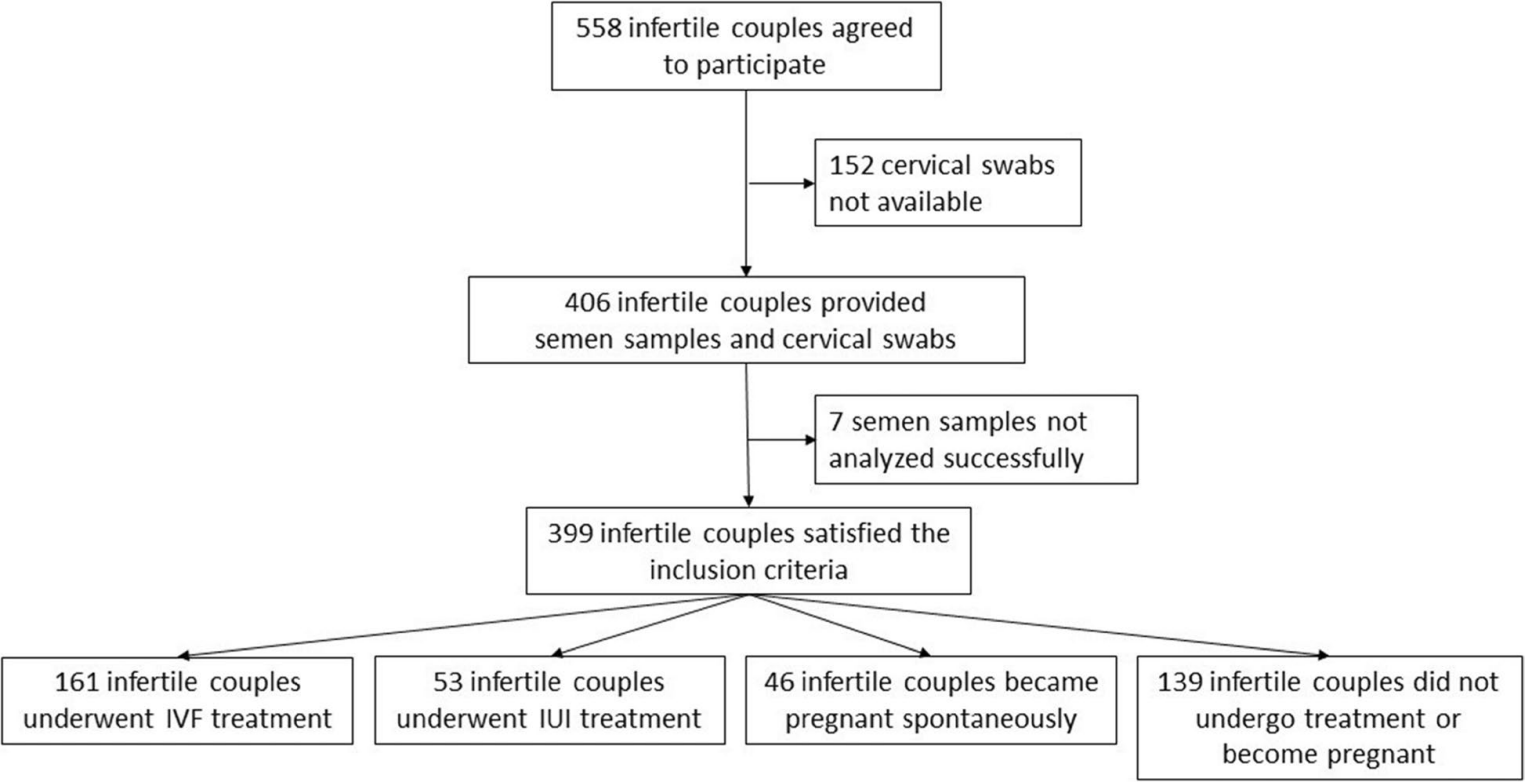
Study flowchart - couples treated for infertility

Individuals considered eligible to participate in the first part of the study were male partners from couples treated for infertility and potential sperm donors who provided both semen samples and penile swabs (Fig. 1). For the second part of the study, where fertility outcomes were evaluated, we included couples treated for infertility who provided both a semen sample and a cervical swab (Fig. 2). All samples were collected between July 2013 and November 2016 together with other samplings. Samples were collected at two Czech fertility centers (Fertimed Ltd., Olomouc and Arleta IVF Ltd., Kostelec nad Orlici) and at SpermBank International Ltd., Olomouc, which operate in the same region. The demographic characteristics of tested subjects were considered comparable.

Inclusion criteria for men from infertile couples were: duration of infertility greater than 1 year and infertility due to various causes. Inclusion criteria for women from infertile couples were: duration of infertility greater than 1 year, infertility due to various causes, and age between 18 and 49 years.

Potential sperm donors were tested according to the criteria specified in the European Commission Directive 2004/23/ES and the Czech Directive 296/2008. HPV testing was performed simultaneously.

### Collection of semen samples and penile swabs from potential sperm donors

Penile swabs and semen samples were collected from a cohort of 328 IM and 97 potential SD (Fig. 1). Participants self-collected penile swab samples by wiping a dry cotton swab at least three times around the coronal sulcus and the top of the glans. Each swab was then rinsed in 0.5 ml of cobas*®* PCR Cell Collection Media (Roche Diagnostics GmBH, Mannheim, Germany). Semen samples were subsequently obtained by masturbation after 3 to 5 days of sexual abstinence. After liquefaction of the ejaculate at room temperature, at least 0.1 ml of ejaculate was placed in 20 ml of cobas*®* PCR Cell Collection Media (Roche Diagnostics GmBH, Mannheim, Germany). The media were transported and stored at room temperature until testing according to the manufacturer’s recommendations for cervical samples. Semen analysis (volume, pH, viscosity, sperm concentration, motility, and normal morphology) was performed according to World Health Organization guidelines [16].

### Collection of cervical swabs and semen samples from couples treated for infertility

Cervical swabs and semen samples were collected from 399 female and male partners treated for infertility (Fig. 2). Cervical swabs were taken from women before planned intrauterine insemination (IUI), in vitro fertilization (IVF) or intracytoplasmic injection (ICSI) treatment. Cervical brushes were rinsed in 20 ml of cobas*®* PCR Cell Collection Media (Roche Diagnostics GmBH, Mannheim, Germany) and transported and stored at room temperature according to the manufacturer’s recommendations for cervical samples. Semen samples were obtained as described above.

For infertility treatment, spermatozoa were separated either by swim-up or density gradient techniques and used in either IUI, IVF or IVF + ICSI techniques. The number of pregnancies (gestational sac and fetal heart beats proven by ultrasound within weeks six to nine of pregnancy) and abortions (blighted ovum or miscarriage) was evaluated, relative to HPV status, in women who underwent IVF/IVF + ICSI (*n* = 161), IUI (*n* = 53) within 6 months after sampling or who became pregnant spontaneously (*n* = 46) within 6 months after sampling without any ART. Biochemical pregnancies (just positive hCG) were not counted as abortions. For IVF/IVF + ICSI, only embryo transfers (ETs) with one or two fresh embryos developed from the woman’s own oocytes fertilized by the spermatozoa of male partner were included.

### HPV DNA detection

Cervical swabs and semen samples were tested for HPV DNA using the cobas*®* 4800 HPV Test (Roche Diagnostics GmBH, Mannheim, Germany) according to the manufacturer’s recommendations for cervical sample management [17]. After analysis, DNA extracted using cobas × 480 was used for HPV DNA detection and genotyping using the PapilloCheck*®* HPV-Screening kit (Greiner Bio-One, Frickenhausen, Germany) [18] as described previously [19]. DNA from penile swabs was isolated using the QIAamp*®* DNA detection by Micro kit (Qiagen, Hilden, Germany) and then tested for HPV PapilloCheck*®* HPV-Screening kit [18].

**Fig. 3:**
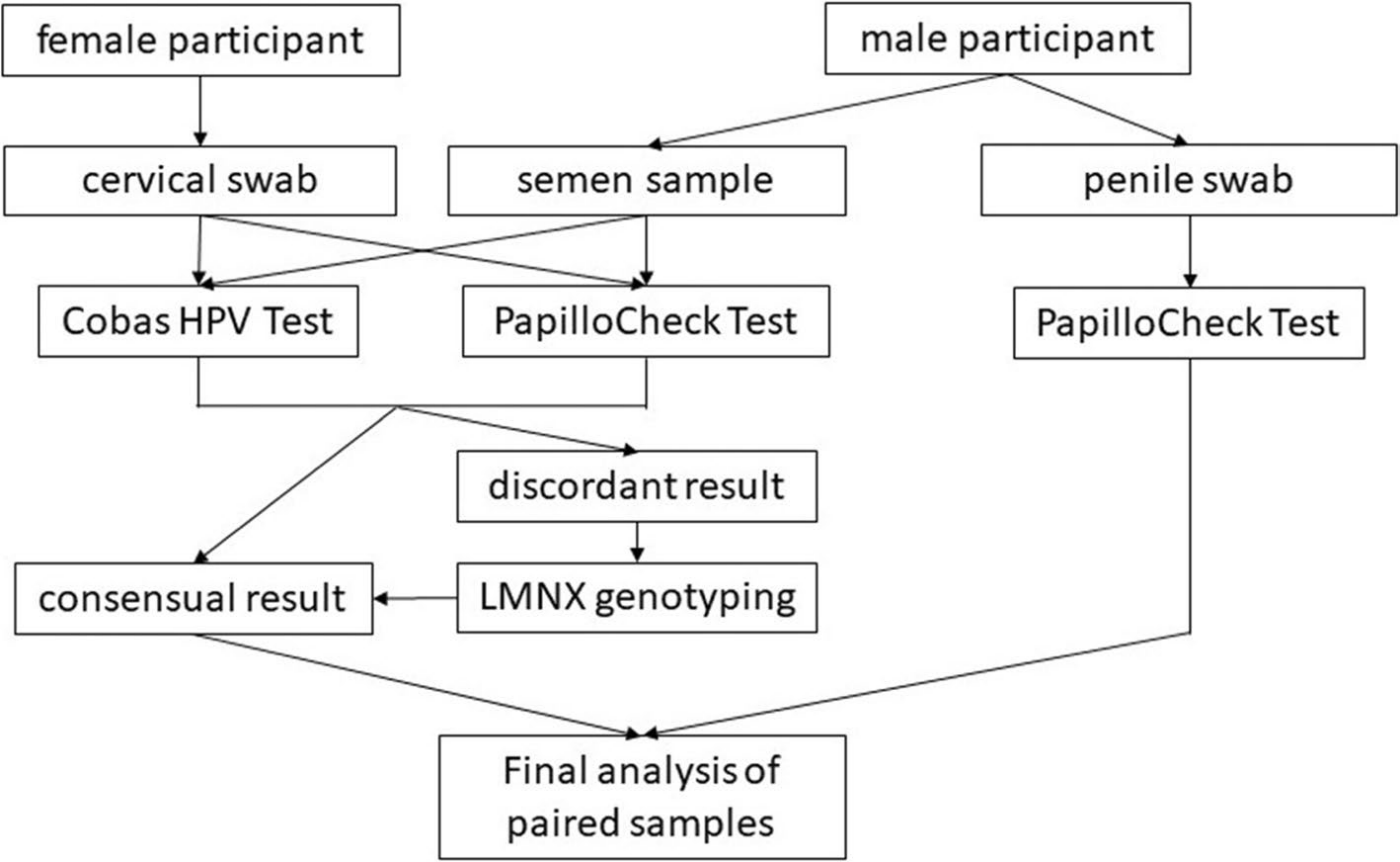
Methods used for HPV detection in different sample types

The cobas*®* 4800 HPV Test and PapilloCheck*®* HPV-Screening gave discordant results for 40 of the 425 semen samples examined in this study. These samples were further analyzed using the LMNX Genotyping Kit HPV GP (Diassay, Rijswijk, The Netherlands) [20] (Fig. 3).

#### Other sexually transmitted diseases detection

The presence of *Chlamydia trachomatis*, *Ureaplasma*, *Mycoplasma genitalium* and *Mycoplasma hominis* was tested in all HPV positive semen samples using the Сhlamydia trachomatis/Ureaplasma/M.genitalium/M.hominis Real-TM kit (Sacace Biotechnologies, Como, Italy) [20]. DNA was extracted from a 1 ml semen sample in cobas*®* PCR Cell Collection Media using QIAamp DNA Micro kit (Qiagen, Hilden, Germany) and eluted into 50 μl of DEPC treated water (Sigma-Aldrich, St. Louis, USA).

### Statistical analysis

The statistical software R (version 3.5.0; R Core Team, *R Foundation for Statistical Computing* [http://www.r-project.org]) was used for data evaluation. The associations between hrHPV positivity and semen parameters, fertility outcomes, and socio-behavioral and clinical characteristics were assessed using Fisher’s exact test, Pearson’s chi-squared test, or the Wilcoxon exact test as appropriate. Data from questionnaires were analysed only if available. Multivariate analysis was performed using a multivariate logistic regression model with adjustment to categorized age. *P*-value ≤0.05 was considered statistically significant. Only infertile couples who underwent ART within 6 months after sampling or became pregnant spontaneously within 6 months after sampling were included in the statistical analysis of fertility outcomes (*n* = 260).

## Results

### HPV positivity rates in potential sperm donors and men from infertile couples

The median age of men from infertile couples (IM; *n* = 328) and potential sperm donors (SD; *n* = 97) were 35 years (range, 22–62 years) and 23 years (range, 19–36 years, *P* < 0.001), respectively. We detected the DNA of at least one of 18 hrHPV genotypes or 6 lrHPV genotypes in 41.9% (178/425) of total samples, 38.1% (37/97) of SD samples, and 43.0% (141/328) of IM samples. HrHPV genotypes were detected in 28.9% (28/97) of SD samples and 35.1% (115/328) of IM samples (*P* = 0.312). LrHPV genotypes were detected in 16.5% (16/97) of SD samples and 15.2% (50/328) of IM samples (*P* = 0.889). The most prevalent hrHPV types detected in semen samples were HPV52, HPV56 and HPV66 (2/6) in sperm donors and HPV53 (9/39) in men from infertile couples. The most prevalent hrHPV type detected in penile swabs was HPV51 (9/26) in sperm donors and HPV16 (27/106) in men from infertile couples. In couples treated for infertility the most prevalent hrHPV genotype was HPV53 (10/67) in male partner and HPV16 (14/35) in female partner.

Men with hrHPV positive penile swabs and/or semen samples from both groups had more sexual partners than those who were hrHPV negative (*P* < 0.001 and *P* = 0.021). The frequency of hrHPV positive penile swabs was higher among IM who had previously had reproductive tract surgery than among IM who had not underwent any reproductive surgery (53.8% vs. 30.4%, *P* = 0.026) (Additional file 1).

Only 2 of the 425 (0.47%) tested men were vaccinated against HPV (Cervarix or Silgard/Gardasil). One of these men was positive for HPV51 based on a penile swab. This genotype is not targeted by any vaccine.

### hrHPV positivity in semen samples and semen quality

**Fig. 4:**
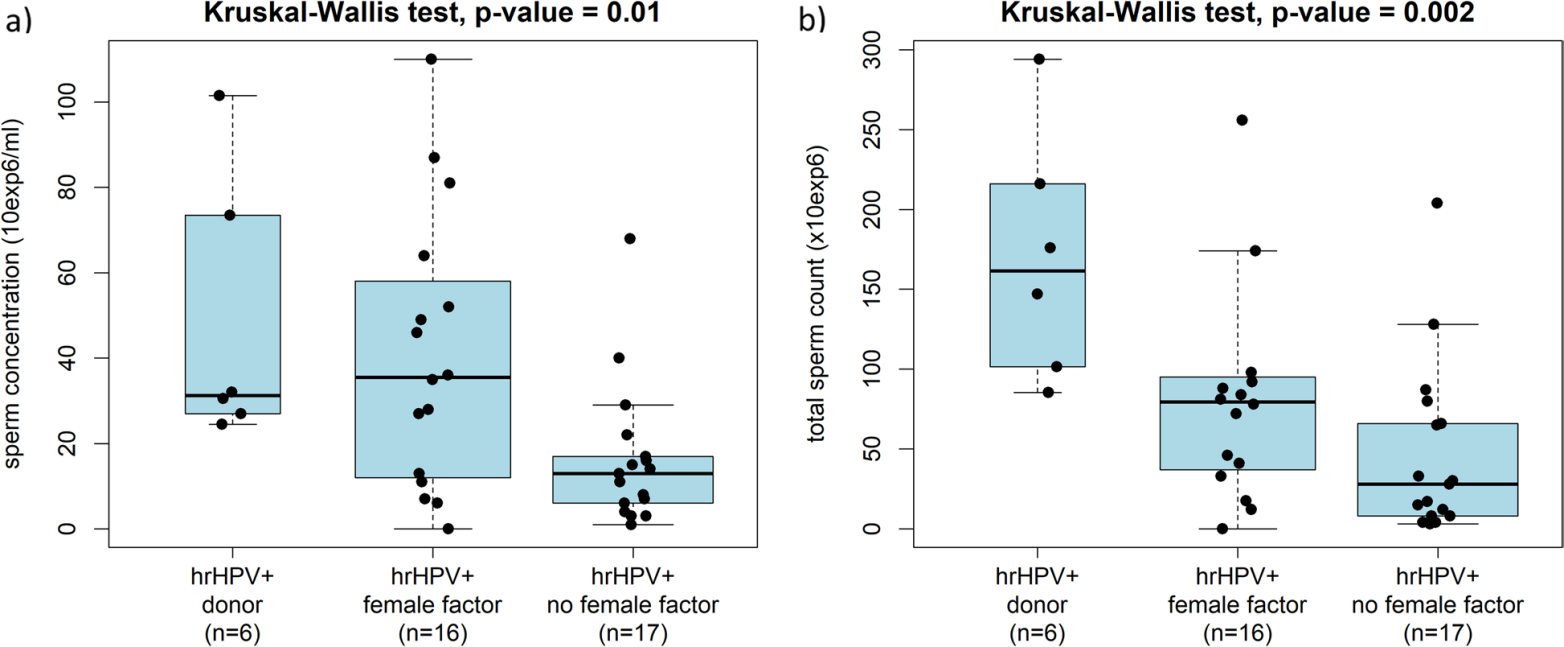
Sperm concentration (**a**) and total sperm count (**b**) in men with hrHPV positive semen sample in sperm donors, men from infertile couples with female factor infertility and men from infertile couples without female factor infertility. Men with genetic factor of infertility were excluded from analysis

IM with hrHPV positive semen samples had lower average semen volume (median volume 2 ml vs. 3 ml, *P* = 0.002), sperm concentration (median concentration 13 × 10^6^/ml vs. 26 × 10^6^/ml, *P* = 0.020), and total sperm count (median count 33 × 10^6^ vs. 71.8 × 10^6^, *P* = 0.004) than those with hrHPV negative samples. No association was identified between hrHPV positivity in semen samples and semen quality in potential SD (Table 1). Among males with hrHPV positive semen samples, sperm concentrations and total sperm counts were significantly lower in IM from couples with no female factor of infertility than in sperm donors and IM from couples with a female factor of infertility (*P* = 0.01 and *P* = 0.002, Fig. 4). Semen quality in men with hrHPV+/HPV+ semen sample was not affected by infection with *Chlamydia trachomatis*, *Ureaplasma*, *Mycoplasma genitalium*, or *Mycoplasma hominis* (Additional file 2). All semen parameters in SD and IM differed significantly (Additional file 3).

**Table 1:** Evaluation of semen parameters in relation to hrHPV result

**Factor**	**Semen samples**						***P*-value (total in semen samples)**	**Penile swabs**						***P*-value (total in penile swabs)**
	**Potential sperm donors**			**Men from infertile couples**				**Potential sperm donors**			**Men from infertile couples**			
	hrHPV+/ hrHPV- (*n*)	**hrHPV+/ hrHPV- (median)**	***P*-value**	hrHPV+/ hrHPV- (*n*)	**hrHPV+/ hrHPV- (median)**	***P*-value**		hrHPV+/ hrHPV- (*n*)	**hrHPV+/ hrHPV- (median)**	***P*-value**	hrHPV+/ hrHPV- (*n*)	**hrHPV+/ hrHPV- (median)**	***P*-value**	
Age	6/91	26/23	0.404	39/289	36/35	0.812	0.431	26/71	23/23	0.537	106/222	35/35	0.898	0.422
Semen pH	6/91	7.3/7.4	0.438	39/289	8/8	0.574	0.519	26/71	7.4/7.4	0.454	106/222	8/8	0.156	0.173
Semen volume (ml)	6/91	4.8/3	0.167	39/289	2/3	**0.002**	**0.009**	26/71	3.6/3	**0.015**	106/222	3/3	0.200	0.908
Sperm concentration (×10^6^/ml)	6/91	31.2/56.5	0.549	39/288	13/26	**0.020**	**0.009**	26/71	46.8/58.5	0.883	106/221	23.5/25	0.286	0.251
Total sperm count (×10^6^)	6/91	161.5/167.2	0.952	39/288	33/71.8	**0.004**	**0.005**	26/71	189.2/165.1	0.297	106/221	62.5/71.5	0.182	0.462
Motility (%)	6/90	50/51.5	0.970	39/288	53/58.5	0.109	0.181	26/70	52/51.5	0.201	106/221	57/58	0.580	0.884
Progressive motility (%)	6/90	35.5/39	0.958	39/288	34/35	0.136	0.110	26/70	36/39	0.923	106/221	35/35	0.515	0.456
Normal sperm morphology (%)	6/90	10.5/11	0.660	39/288	5/7	0.233	0.095	26/70	10.5/11	0.620	106/221	6.5/7	0.814	0.582
Number of sexual partners	6/88	7/3	0.053	33/276	7/5	0.143	**0.021**	26/68	6/3	**0.008**	99/210	6/4	**0.003**	**< 0.001**
Age of current sexual partner	4/49	24/25	0.827	37/281	32/32	0.700	0.886	14/39	25/24	0.940	104/214	31/32	0.226	0.628

Statistically significant data (*P*-value < 0.05) are shown in bold

#### Comparison of HPV positivity in semen samples and penile swabs

Of the 178 HPV positive men, 55 (30.9%) had a positive semen sample, 169 (94.9%) had a positive penile swab, and 46 (25.8%) were positive in both samples (Table 2). Penile swabs were more frequently HPV positive than semen samples in both IM (32.3% vs. 11.9%, *P* < 0.001 for hrHPV; 14.9% vs. 4.88% [16/328], *P <* 0.001 for lrHPV) and SD (26.8% vs. 6.2%, *P* = 0.006 for hrHPV; 15.5% vs. 1.03%, *P* = 0.008 for lrHPV, Table 2). There was no association found between hrHPV positivity detected in penile swabs and semen quality (Table 1) and between penile swabs/semen sample lrHPV infection and semen quality. Therefore, when evaluating reproductive outcomes, we focused on hrHPV detection in semen samples only.

**Table 2:** Comparison of HPV positivity in semen samples and penile swabs

	**Potential sperm donors** (*n* = 97)		**Men from infertile couples** (*n* = 328)		***P*-value** (hrHPV)	***P*-value** (hrHPV+lrHPV)
	hrHPV	hrHPV+lrHPV	hrHPV	hrHPV+lrHPV		
S+/PS+	4 (4.12%)	5 (5.15%)	30 (9.15%)	41 (12.5%)	0.165	0.063
S+/PS-	2 (2.06%)	2 (2.06%)	9 (2.74%)	7 (2.13%)	0.994	1.000
S−/PS+	22 (22.68%)	30 (30.93%)	76 (23.17%)	93 (28.35%)	1.000	0.716
S−/PS-	69 (71.13%)	60 (61.86%)	213 (64.94%)	187 (57.01%)	0.311	0.464

HrHPV includes HPV 16, 18, 31, 33, 35, 39, 45, 51, 52, 53, 56, 58, 59, 66, 68, 70, 73, and 82 genotypes

LrHPV includes HPV6, 11, 40, 42, 43, 44/55

*S* Semen samples, *PS* Penile swabs

### HPV positivity and fertility outcomes

The median ages of female and male partners from infertile couples (*n* = 399) were 33 years (range: 20–45 years) and 35 years (range: 21–44 years), respectively. HrHPV genotypes were detected in 16.3% (67/399) of women and 8.77% (35/399) of men from infertile couples (*P* = 0.001). Both partners were hrHPV positive in 2.26% (9/399) of infertile couples. Pregnancy rates in couples treated with IVF (98/161, 60.9%) and couples treated with IUI (27/53, 50.9%) were comparable (*P* = 0.267). The abortion rates in spontaneously pregnant women (5/46, 10.9%), couples treated with IVF (6/98, 6.12%), and couples treated with IUI (1/27, 3.70%) did not differ significantly (*P* = 0.489).

No associations between hrHPV infection of male, female, or both partners and lower pregnancy rates or higher abortion rates were identified in couples treated for infertility, regardless of the method of conception (Table 3). Among infertile couples with unexplained infertility, no women with a hrHPV+ male partner became pregnant spontaneously (0/5), whereas 43.2% of women (16/37) with a hrHPV- male partner became pregnant spontaneously (*P* = 0.138). The absence of association between hrHPV positivity and fertility outcomes was confirmed by multivariate analysis (Additional files 4, 5, and 6).

**Table 3:** Fertility outcomes in infertile couples according to hrHPV status

	**IVF (*n* = 161)**				**IUI (*n* = 53)**				**Spontaneous pregnancy (*n* = 46)**			
	**Pregnancy (%)**	***P*-value**	**Abortion (%)**	***P*-value**	**Pregnancy (%)**	***P*-value**	**Abortion (%)**	***P*-value**	**Pregnancy (%)**	***P*-value**	**Abortion (%)**	***P*-value**
hrHPV+ male												
Yes	10/11 (90.9%)	0.051	1/10 (10%)	0.485	3/6 (50%)	1.000	0/3 (0%)	1.000	2/18 (11.1%)^a^	0.250	0/2 (0%)	1.000
No	88/150 (58.7%)		5/88 (5.7%)		24/47 (51.1%)		1/24 (4.2%)		44/167 (26.3%)^b^		5/44 (11.4%)	
hrHPV+ female												
Yes	16/20 (80%)	0.103	1/16 (6.2%)	1.000	1/6 (16.7%)	0.100	0/1 (0%)	1.000	9/40 (22.5%)^a^	0.854	0/9 (0%)	0.566
No	82/141 (58.2%)		5/82 (6.1%)		26/47 (55.3%)		1/26 (3.8%)		37/145 (25.5%)^b^		5/37 (13.5%)	
hrHPV+ couple												
Yes	2/3 (66.7%)	1.000	0/2 (0%)	1.000	0/1 (0%)	0.491	0/0 (0%)	1.000	0/5 (0%)^a^	0.436	0/0 (0%)	NA
No	96/158 (60.8%)		6/96 (6.2%)		27/52 (51.9%)		1/27 (3.7%)		46/180 (25.6%)^b^		5/46 (10.9%)	

HrHPV includes HPV 16, 18, 31, 33, 35, 39, 45, 51, 52, 53, 56, 58, 59, 66, 68, 70, 73, and 82 genotypes

The *P*-value was calculated using Pearson’s chi-square test

^a^All hrHPV positive male/female partners or whole couple not treated by ART (IVF or IUI)

^b^All hrHPV negative male/female partners or whole couple not treated by ART (IVF or IUI)

## Discussion

We found a high prevalence of hrHPV infection in both groups of a large study cohort comprising male partners from infertile couples and potential sperm donors, (35.1 and 28.9%), with significantly higher hrHPV positivity in penile swabs than semen samples in both IM (32.3% vs. 11.9%, *P* < 0.001) and SD (26.8% vs. 6.2%, *P* = 0.006). The hrHPV prevalence in semen samples from IM in our study cohort was lower than the pooled hrHPV prevalence reported in the meta-analysis of Laprise et al. [21] (11.9% vs. 16%; *P* = 0.81), and that for SD was significantly lower (6.2% vs. 17.4%; *P* = 0.01).

We demonstrated a significant association between hrHPV positive semen samples in IM and lower semen volume, sperm concentration, and total sperm count. In keeping with these findings, two previous publications reported lower sperm counts in men with positive semen samples [22, 23]. This could be due to HPV-driven DNA fragmentation in spermatozoa, which leads to apoptosis [24, 25]. In several studies, HPV semen infection was found to be associated with significantly lower sperm motility [8, 22, 24, 26–30] due to an increase in the number of anti-sperm antibodies binding on the sperm surface [27, 28]. Reduced motility in men with HPV positive semen samples was also observed in this study, but the association was not statistically significant (*P* = 0.109).

In vitro experiments showed that binding of HPV to sperm head affects sperm parameters, reduces the penetration rate of HPV positive sperm, and could also transfer HPV virions to the oocyte [9] and induce stage-specific maturation arrest and apoptosis in HPV-infected embryos [31]. To date, Garolla et al. [28] have reported significantly reduced spontaneous pregnancy rates in infertile couples without known infertility factors (HPV+ vs. HPV-, 0% vs. 8.1%, *P* = 0.04) [28]. Similarly, Depuydt et al. showed reduced clinical pregnancy rates in women receiving inseminations with HPV positive semen [32, 33]. Our results indicated that semen HPV positivity in men from infertile couples with unexplained infertility was associated with a reduced pregnancy rate, but the difference was not statistically significant (HPV+ vs. HPV-, 0% vs. 43.2%, *P* = 0.138). This could have been caused by relatively few couples with unexplained infertility (*n* = 42) in the group without ART treatment.

Garolla et al. [28] also reported that HPV positivity in the male partner reduced ART success rate in both IUI (HPV+ vs. HPV-, 9.5% vs. 20%, *P* = 0.449) and ISCI (HPV+ vs. HPV-, 18.2% vs. 40.8%, *P* = 0.032) [28]. A similar correlation between reduced pregnancy rates and HPV DNA positivity in semen samples from male partners in infertile couples treated by IVF was reported by Perino et al. [15] (66.7 vs. 15%, *P <* 0.01). We found no association between HPV DNA positivity in semen samples and reduced ART success rates, which could be due to the small number of couples using ART in the groups with unexplained infertility. Moreover, we observed no significant association between HPV positivity in the female partner or both partners of infertile couples and reduced pregnancy rates.

An in vitro study indicated that HPV could negatively influence early embryo development, which could increase abortion rates in ART-treated couples where the male partner is HPV positive [9]. This conclusion was supported by the results of two clinical studies [15, 28] in which infertile couples with a HPV positive male partner had higher abortion rates than those with a HPV negative male partner (62.5% [5/8] vs. 16.7% [11/66], *P <* 0.05 and 66.7% [4/6] vs. 15% [9/60], *P <* 0.01). In our study, no association between hrHPV infection of male, female or both partners and higher abortion rate was identified in ART-treated couples or in couples with spontaneous pregnancy.

## Conclusions

In conclusion, we have demonstrated a high prevalence of hrHPV infection in both potential sperm donors and men from infertile couples, with higher hrHPV prevalence in penile swabs compared to semen samples. HrHPV positivity in semen samples was significantly associated with reduction in semen parameters. Although we found no significant association between the presence of hrHPV DNA in semen and fertility outcomes, the data suggest that HPV infection influences male fertility and probably reduces spontaneous pregnancy rates. HPV infection probably does not affect the pregnancy rates after ART, nevertheless could adversely affect the success of spontaneous pregnancies.

## Supplementary Information

**Additional file 1. **Evaluation of questionnaires in relation to hrHPV results. *These two potential sperm donors underwent a circumcision. Sexually transmitted disease includes *Chlamydia trachomatis*, HPV, *Trichomonas vaginalis*, *Neisseria gonorhaea*, HSV, *Ureaplasma*, *Mycoplasma* and Cytomegalovirus. Statistically significant data (*P*-value < 0.05) are shown in bold.

**Additional file 2.** Other sexually transmitted infections in HPV positive semen samples. MO-microorganism.

**Additional file 3. **Comparison of semen parameters in potential sperm donors and in men from infertile couples. Statistically significant data (*P*-value < 0.05) are shown in bold.

**Additional file 4. **Fertility outcomes in infertile couples treated by IVF according to cause of infertility, age and hrHPV status. The *P*-value was calculated using Fisher’s exact test (*) or multivariate logistic regression model with categorized age as adjusting factor (**). Statistically significant data (*P*-value < 0.05) are shown in bold. CI- confidence interval, NA- not available, OR- odds ratio.

**Additional file 5. **Fertility outcomes in infertile couples treated by IUI according to cause of infertility, age and hrHPV status. The *P*-value was calculated using Fisher’s exact test (*) or multivariate logistic regression model with categorized age as adjusting factor (**). Statistically significant data (*P*-value < 0.05) are shown in bold. CI- confidence interval, NA- not available, OR- odds ratio.

**Additional file 6. **Fertility outcomes in infertile couples in which women become pregnant spontaneously according to cause of infertility, age and hrHPV status. The *P*-value was calculated using Fisher’s exact test (*) or multivariate logistic regression model with categorized age as adjusting factor (**). Statistically significant data (*P*-value < 0.05) are shown in bold. CI- confidence interval, NA- not available, OR- odds ratio.

## Data Availability

The datasets used and analyzed during the current study are available from the corresponding author on reasonable request.

## Abbreviations

ART: Assisted reproduction treatment
HPV: Human papillomavirus
hrHPV: High risk human papillomavirus
IM: Male partners of infertile couples
ICSI: Intracytoplasmic injection
IUI: Intrauterine insemination
IVF: In vitro fertilization
SD: Potential sperm donor
